# Seafood allergy comprises IgE-defined molecular sensitization endotypes associated with distinct clinical severity profiles

**DOI:** 10.1016/j.jacig.2026.100787

**Published:** 2026-09-21

**Authors:** Enrico Scala, Damiano Abeni, Elisabetta Caprini, Ignazio Brusca, Maria Barrale, Valerio Pravettoni, Chiara Casale, Mauro Giani, Lorenzo Cecchi, Danilo Villalta, Riccardo Asero

**Affiliations:** a Fondazione Luigi Maria Monti, IDI-IRCCS, Rome, Italy; b U.O.C of Clinical Pathology, Buccheri La Ferla Hospital, Palermo, Italy; c SC Medicina - Immunologia e Allergologia, Fondazione IRCCS Ca' Granda Ospedale Maggiore Policlinico, Milan, Italy; d Allergy and Clinical Immunology Unit, San Giovanni di Dio Hospital, Florence, Italy; e S.C. di Immunologia e Allergologia di Laboratorio, PO S. Maria degli Angeli, Pordenone, Italy; f Ambulatorio di Allergologia, Clinica San Carlo, Paderno Dugnano, Milan, Italy

**Keywords:** Seafood allergy, component-resolved diagnosis, multiplex molecular diagnostics, IgE sensitization, molecular sensitization profiles, reaction severity, parvalbumin, tropomyosin, arginine kinase, Ani s 1

## Abstract

**Background:**

Seafood allergy is characterized by heterogeneous IgE sensitization to distinct molecular components, but the clinical relevance of these profiles remains incompletely defined. Multiplex molecular diagnostics may help identify molecular endotypes and refine risk stratification.

**Objective:**

This study aimed to characterize IgE-defined molecular sensitization patterns to major seafood allergens and assess their associations with clinical reaction severity.

**Methods:**

In this multicenter prospective study (January 2021 to December 2025), 8,040 consecutive patients screened with the ALEX^2^ multiplex immunoassay were evaluated; 787 (9.8%) were sensitized to ≥1 seafood-derived molecule. Sensitization to parvalbumin, tropomyosin, Ani s 1, arginine kinase, and other muscle-related proteins was analyzed in relation to prospectively collected clinical phenotypes (mild, moderate, and severe). Venn analysis, principal component analysis, and unsupervised hierarchical clustering explored molecular patterns. Multivariable binary logistic regression models were fitted for each severity category versus all others, adjusting for age and sex. Associations are reported as odds ratios (ORs) with 95% CIs.

**Results:**

Tropomyosin (45%), arginine kinase (37%), parvalbumin (24%), and Ani s 1 (12%) were the most prevalent sensitizations and showed limited overlap, delineating largely distinct molecular profiles. Hierarchical clustering and 3-dimensional principal component analysis confirmed the separation of major components in multivariate space, with distinct loading patterns. In adjusted analyses, parvalbumin (adjusted odds ratio [OR_adj], 3.7) and Ani s 1 (OR_adj, 2.7) were associated with severe reactions, tropomyosin with moderate reactions (OR_adj, 2.0), and arginine kinase with mild phenotypes (OR_adj, 5.3).

**Conclusions:**

Major seafood allergens define largely distinct and minimally overlapping sensitization patterns that are statistically associated with specific clinical phenotypes.

Current international guidelines do not recommend the routine use of allergen multiplex arrays in the diagnostic workup of food allergy, largely owing to concerns regarding standardization, cost-effectiveness, and limited direct evidence linking molecular sensitization profiles to clinical outcomes.^1^ Nevertheless, multiplex molecular diagnostics provide a unique opportunity to disentangle complex sensitization patterns that are insufficiently captured by conventional extract-based assays or by sequential single-component testing, which is highly relevant for diagnostic purposes but not fully replicable through multiplex technologies.^2^ By enabling the simultaneous assessment of IgE reactivity to multiple purified molecules within the same individual, allergen microarrays facilitate identification of both cosensitization clusters and mutually exclusive molecular profiles.^3^

In seafood allergy, patients frequently exhibit heterogeneous IgE responses directed against structurally and functionally distinct allergens derived from fish, crustaceans, molluscs, or parasites, including tropomyosin, parvalbumin, Ani s 1, and arginine kinase.^4^, ^5^ Conventional diagnostic strategies may obscure the underlying variability in individual IgE sensitization profiles, potentially leading to misclassification of risk and inappropriate dietary restrictions. Molecular allergology enables refined characterization of sensitization patterns and may support personalized management and risk-discriminative profiling.^6^, ^7^

The present prospective observational study was designed to investigate the prevalence, the degree of overlap, and the clinical correlates of sensitization to major seafood allergens. We hypothesized that seafood allergy is not merely heterogeneous but organized into discrete IgE-defined molecular endotypes that map onto differential clinical risk trajectories, thereby challenging extract-based diagnostic paradigms.

## Methods

### Study design and population

This multicenter prospective observational study was conducted from January 2021 to December 2025 across 3 specialized allergy centers in Northern (Lombardy and Friuli Venezia Giulia), Central (Lazio and Tuscany), and Southern Italy (Sicily). Consecutive patients undergoing molecular allergology diagnostics with ALEX^2^ were screened. Written informed consent was obtained from all participants, and the protocol was approved by the institutional review boards of participating centers. The research was conducted in accordance with ethical standards outlined in the World Medical Association Declaration of Helsinki. The study protocol underwent review and approval by the IDI-IRCCS’s committee, with approval number IDI-IRCCS CE | 492/1.

### Molecular allergy testing

Serum allergen-specific IgE profiles were assessed using the ALEX^2^ multiplex immunoassay (MacroArray Diagnostics, Vienna, Austria), which enables simultaneous detection of IgE antibodies against a broad panel of seafood- and invertebrate-derived allergens. The panel included *Anisakis simplex* components, namely Ani s 1 (a Kunitz-type serine protease inhibitor) and Ani s 3 (tropomyosin); tropomyosins from mite (Der p 10, Blo t 10), crustacean (Pen m 1), and insect sources (Per a 7); and arginine kinases from cockroach (Bla g 9), mite (Der p 20), and shrimp (Pen m 2). Fish parvalbumins from multiple species were also included (Clu h 1, Cyp c 1, Gad m 1, Raj c α-parvalbumin, Sal s 1, Sco s 1, Thu a 1, and Xip g 1). Additional crustacean muscle proteins comprised myosin light chain (Pen m 3), troponin C (Cra c 6), β-enolase/aldolase (Gad m 2/3), and sarcoplasmic calcium-binding protein (CBP) (Pen m 4). Sensitization was defined as specific IgE concentrations exceeding the manufacturer’s cutoff (>0.3 kUA/L).

### Clinical data collection

Demographic and clinical variables were prospectively recorded in a dedicated electronic database at the initial visit, prior to availability of serological results, ensuring blinding to molecular data. Seafood-related allergic reactions were categorized according to established criteria^8^ as mild (isolated oral symptoms), moderate (urticaria, angioedema, or asthma without systemic involvement), or severe (anaphylaxis involving ≥2 organ systems, hypotension, or syncope). Clinical phenotypes were history based and not challenge confirmed.

### Statistical analysis

Descriptive statistics were used to summarize demographic, clinical, and molecular data (means ± SDs for continuous variables; counts and percentages for categorical variables). Between-group comparisons were performed using chi-squared or Fisher exact tests for categorical variables and the Mann-Whitney *U* test for continuous variables when distributional assumptions were not satisfied.

Clinical phenotypes were classified into 4 mutually exclusive categories (absent, mild, moderate, and severe). Although these categories can be ranked by severity, they were analyzed as distinct clinical conditions rather than as stages along a progression continuum. Given the nonproportional distribution of severity categories in exploratory analyses and the objective of estimating independent risk profiles for each clinical severity class, separate multivariable binary logistic regression models were employed rather than an ordinal proportional-odds or multinomial approach. For each severity category, a dedicated model was fitted comparing that category with all remaining patients (mild vs others, moderate vs others, and severe vs others), with adjustment for age and sex. All sensitization variables were entered simultaneously in each model. Multicollinearity was assessed using variance inflation factors. Observed variance inflation factor values ranged from 1.015 to 1.274, indicating no evidence of problematic collinearity among predictors. The number of outcome events per predictor variable exceeded commonly recommended thresholds (≥10 events per variable), supporting model stability. No evidence of complete or quasiseparation was observed, and all logistic regression models achieved stable maximum likelihood convergence. Model calibration was assessed using the Hosmer–Lemeshow goodness-of-fit test.

Odds ratios (ORs) with 95% CIs were reported for univariate and adjusted analyses. Because the primary hypotheses concerned a predefined set of major seafood allergen components, no formal correction for multiple testing was applied; nevertheless, key associations were robust to conservative multiplicity thresholds. Sensitivity analyses were conducted excluding asymptomatic sensitized individuals to evaluate the robustness of associations across clinically reactive subjects. No missing data were present for the variables included in the analyses among the 787 participants meeting inclusion criteria; all models were therefore fitted on the complete-case dataset.

Unsupervised analyses were performed to explore patterns of molecular and clinical coreactivity. For principal component analysis (PCA), an ordinal/semiquantitative encoding was used (−3 indicating absence of allergen reactivity; 0 indicating sensitization without symptoms; 1, 2, and 3 indicating mild, moderate, and severe reactions, respectively). PCA was applied to the correlation matrix of standardized variables and interpreted as exploratory, acknowledging that the geometry may depend on the chosen encoding. As a robustness check, PCA results were compared with analyses based on sensitization-only coding, yielding consistent qualitative structure. Hierarchical clustering was performed on both allergen components and subjects using Pearson correlation distance as the similarity metric and complete linkage as the agglomeration rule.

All tests were two-sided, and a *P* value < .05 was considered statistically significant. Analyses were performed using SPSS (version 27.0; IBM Corp, Armonk, NY). Data visualizations were produced with GraphPad Prism (version 9.0; GraphPad Software, San Diego, Calif). Unsupervised clustering and PCA were performed using the Genesis platform.^9^ Venn diagrams were generated with VennMaster version 0.38 (Ulm University, Ulm, Germany).^10^ The study is reported in accordance with Strengthening the Reporting of Observational Studies in Epidemiology guidelines for observational studies.

## Results

### Study population

Of 8,040 screened patients, 787 met inclusion criteria (IgE sensitization to ≥1 seafood-derived molecule). The cohort comprised 377 males (47.9%) and 410 females (52.1%), with a mean age of 34 ± 19 years.

### Molecular sensitization profiles

Marked heterogeneity was observed across allergenic components (Table I). Tropomyosin was the most prevalent sensitization (n = 357; 45%), followed by arginine kinase (n = 290; 37%), parvalbumin (n = 189; 24%), and Ani s 1 (n = 94; 12%). Less frequent sensitizations included troponin C (8%), myosin light chain (6%), enolase/aldolase (5%), and sarcoplasmic CBP (2%). Age at sensitization differed across molecular families. Patients sensitized to enolase/aldolase and sarcoplasmic CBP were significantly younger compared with those who were not sensitized, whereas no significant age differences emerged for Ani s 1 or tropomyosin. Sex distribution was overall balanced across most components; however, male sex was independently associated with parvalbumin and troponin C sensitization, whereas no significant sex effect was observed for tropomyosin.

**Table I:** Molecular sensitization profiles among participants (N = 787)

Allergen	N (787)	Percent (%)	Age (mean ± SD) (*P* value)	Male (%)	Female (%)
Ani s 1 (Kunitz serine PI)	94	12	37 ± 22 (ns)	47	53
Arginine kinase	290	37	32 ± 16 (.021)	51	49
Enolase/aldolase	38	5	21 ± 19 (<.001)	60.5	39.5
Myosin light chain	44	6	35 ± 17 (ns)	41	59
Parvalbumin	189	24	31 ± 19 (.013)	56	44 (*P* < .01)
Sarcoplasmic CBP	19	2	21 ± 13 (<.01)	37	63
Tropomyosin	357	45	35 ± 20 (ns)	47	53
Troponin C	65	8	29 ± 17 (.042)	62	38 (*P* = .014)

For each allergenic molecule, the number of sensitized individuals (N) and the corresponding row percentage of the total cohort (n = 787; %) is reported. Age is expressed as means ± SDs and sex as n (%). *P* values are reported as superscripts next to the corresponding variables; “ns” indicates nonsignificant differences. Age was compared using the Mann-Whitney *U* test and sex using chi-squared or Fisher exact tests, as appropriate. All sensitization was determined by serum-specific IgE measurement using the ALEX^2^ multiplex assay.

*PI*, Protease inhibitor.

Venn analysis provides simple counts of pairwise overlap but can be misleading for complex intersection patterns; hierarchical clustering and 3-dimensional PCA more accurately depict relationships among components. Although the Venn suggests limited pairwise overlap (Fig 1, *A*), 3-dimensional PCA showed that sensitization to Ani s 1, tropomyosin, arginine kinase, and parvalbumin occurred predominantly as independent or minimally overlapping events, with these 4 major components occupying relatively distinct regions of multivariate space and exhibiting differential loadings along the principal axes (Fig 1, *B*). Unsupervised hierarchical clustering further revealed a modular organization: a myofibrillar module including tropomyosin, troponin C, myosin light chain, and sarcoplasmic CBP, with arginine kinase clustering within but positioned apart from the other myofibrillar members; a distinct branch where parvalbumin clusters with enolase/aldolase; and an isolated Ani s 1 cluster (Fig 2).

**Fig 1:**
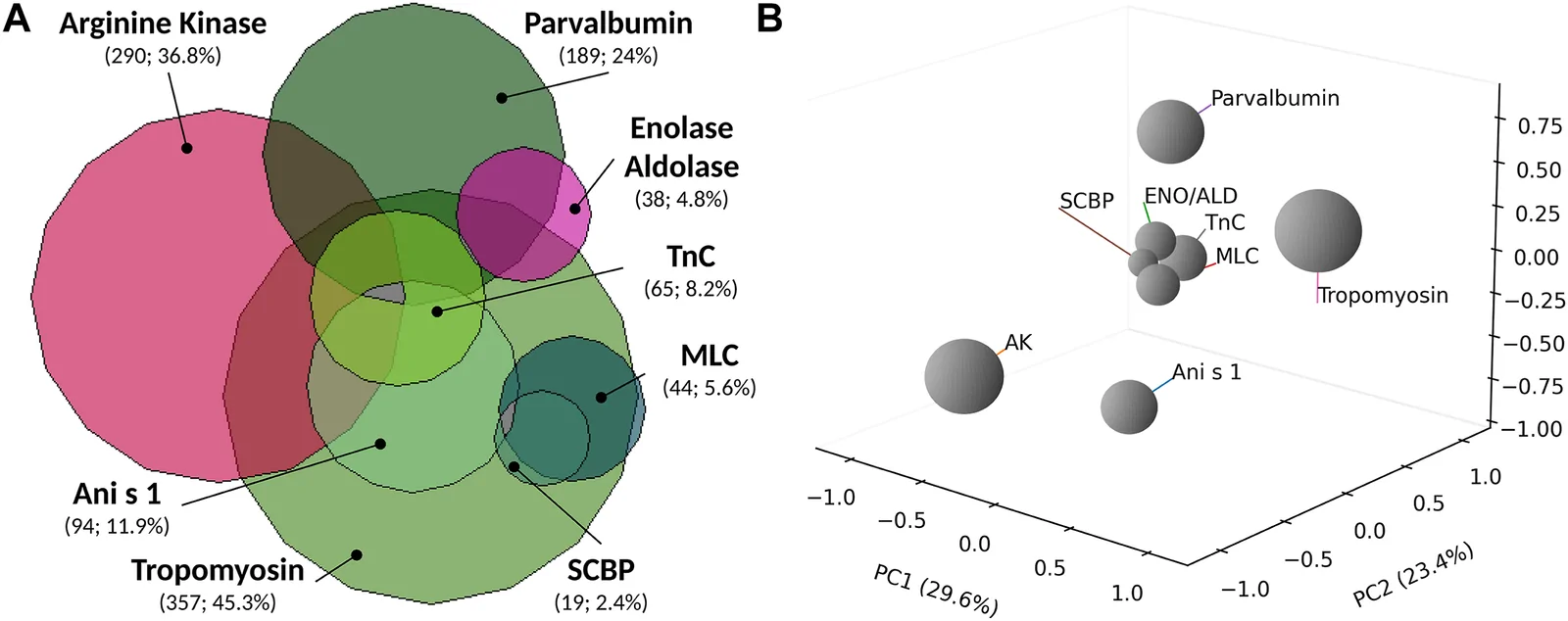
Molecular sensitization patterns to seafood allergens. **(A)** Venn diagram showing overlap of sensitization to Ani s 1, tropomyosin, arginine kinase, and parvalbumin (absolute counts and percentages of the 787 sensitized subjects). **(B)** Three-dimensional PCA plot of the 8 seafood allergen components. Sphere volume is proportional to the prevalence of sensitization in the study population. Spatial separation along the principal component axes (PC1-PC3) reflects differences in IgE reactivity patterns identified by unsupervised analysis. The proportion of variance explained by each principal component is indicated on the corresponding axis; notably, PC3 explains 16.1% of the total variance. *AK*, Arginine kinase; *ENO/ALD*, enolase/aldolase; *TnC*, troponin C; *MLC*, myosin light chain; *SCBP*, sarcoplasmic calcium-binding protein; *PC1*, first principal component; *PC2*, second principal component; *PC3*, third principal component.

**Fig 2:**
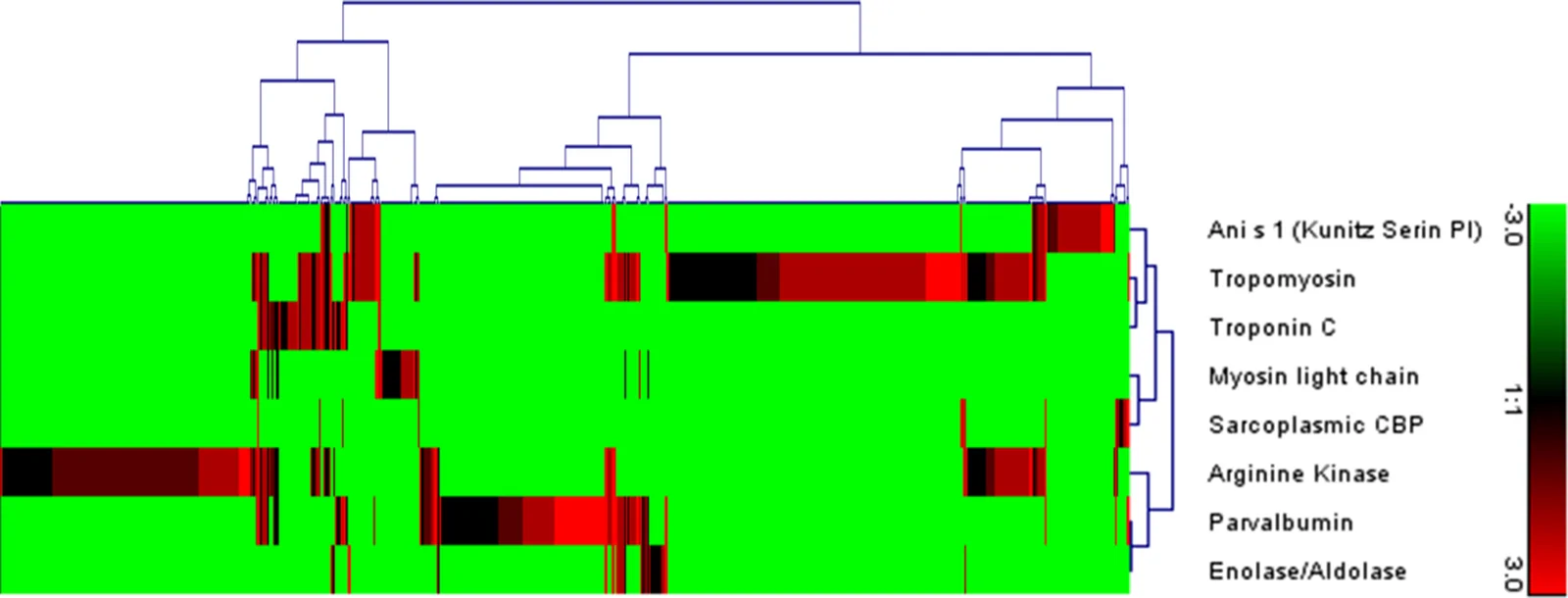
Hierarchical clustering of panallergen reactivity profiles and associated clinical severity. Heatmap with hierarchical clustering of allergen-specific IgE profiles. Rows: allergen components; columns: subjects (n = 787). Color scale: green (no reactivity); black: presence of IgE reactivity without clinical symptoms (asymptomatic sensitization); deep red: mild symptoms (predominantly oral allergy syndrome); red: moderate systemic reactions; bright red: severe reactions, including anaphylaxis as defined in the Methods section. Dendrograms indicate clustering of components and subjects.

### Clinical manifestations

Reaction severity varied by allergen (Table II). Ani s 1 sensitization was strongly associated with moderate reactions (57% of Ani s 1-sensitized individuals) and with severe reactions/anaphylaxis (24%). Tropomyosin sensitization predominantly elicited moderate reactions (53%) with anaphylaxis in 15%. Arginine kinase was mainly linked to mild presentations (42%). Parvalbumin sensitization was associated with a high proportion of moderate (26%) and severe reactions (31%). Although infrequent, sarcoplasmic CBP sensitization was associated with moderate outcomes in 42% and severe reactions in 37% of sensitized individuals. Enolase/aldolase and myosin light chain were more frequently associated with mild phenotypes. Notably, 204 sensitized individuals (n = 204; 25.9%) reported no clinical reaction following seafood ingestion; this asymptomatic subgroup had a mean age of 37 ± 21 years and included 107 females (52.5%). Among asymptomatic subjects, tropomyosin (n = 89; 43.6%), arginine kinase (n = 63; 30.9%), and parvalbumin (n = 56; 27.5%) were the most frequent sensitizing components. These differences were visually represented in the heatmap analysis (Fig 2), which highlighted clusters enriched for predominantly mild or severe symptom profiles. Importantly, clustering appeared to reflect shared structural and biochemical properties rather than uniform clinical risk, with severity gradients emerging at the level of individual components.

**Table II:** Distribution of clinical food allergy reactions according to molecular sensitization profiles

**Allergn**	**Total**	**Food** a**llergy**							
		Absent		Mild reaction		Moderate reaction		Severe reaction	
		**N**	**Row** **N (%)**	**N**	**Row** **N (%)**	**N**	**Row N (%)**	**N**	**Row** **N** **(%)**
Ani s 1	94	7	7	10	11	54	57∗	23	24∗
Arginine kinase	290	63	22	123	42∗	80	28	24	8
Enolase/aldolase	38	12	32	4	11	11	29	11	29
Myosin light chain	44	20	45	2	5	16	36	6	14
Parvalbumin	189	56	30	25	13	49	26	59	31∗
Sarcoplasmic CBP	19	2	11	2	11	8	42	7	37
Tropomyosin	357	89	25	26	7	188	53∗	54	15
Troponin C	65	21	32	5	8	30	46	9	14

For each allergenic molecule, the table reports the total number of sensitized individuals (Total) and, for each clinical category, the number of participants (N) and the row percentage (Row %) calculated on the total number of sensitized individuals for that allergen. Columns are ordered and labeled as follows: absent, mild, moderate, and severe.

∗ Statistical significance for differences in reaction severity across molecular sensitization groups when *P* < .01.

### Association between sensitizations and reaction severity

Univariate analyses identified significant associations between sensitization to Ani s 1, parvalbumin, sarcoplasmic CBP, and tropomyosin and the occurrence of moderate to severe reactions (all *P* < .01; Table III). Arginine kinase showed a strong association with mild reactions in univariate analysis (OR, 7.5; 95% CI, 5.1-10.9; *P* < .001).

**Table III:** Associations between molecular sensitization profiles and severity of clinical reactions

**Allergen**	**Symptoms**	Univariate	***P* value**	Multivariate	***P* value**
		**OR (95% CI)**		**OR_adj (95% CI)**	
Ani s 1 (Kunitz serine PI)	Mild	0.5 (0.2-0.9)	**.016**	0.8 (0.4-1.5)	.444
	Moderate	2.6 (1.7-4.0)	**<.001**	2.0 (1.3-3.3)	**.003**
	Severe	2 (1.2-3.3)	**.009**	2.7 (1.5-4.8)	**.001**
Arginine kinase	Mild	7.5 (5-10.9)	**<.001**	5.3 (3.4-8.4)	**<.001**
	Moderate	0.5 (0.4-0.7)	**<.001**	0.5 (0.4-0.8)	**<.001**
	Severe	0.4 (0.3-0.7)	**<.001**	0.6 (0.4-1.0)	.067
Enolase/aldolase	Mild	0.6 (0.3-1.5)	.311	1.0 (0.4-2.7)	.957
	Moderate	0.6 (0.3-1.2)	.125	0.6 (0.3-1.2)	.158
	Severe	2.7 (1.3-5.5)	**.005**	1.4 (0.6-3.1)	.429
Myosin light chain	Mild	0.2 (0.1-0.8)	**.010**	0.3 (0.1-1.1)	.077
	Moderate	0.8 (0.4-1.6)	.571	0.9 (0.5-1.8)	.792
	Severe	0.9 (0.4-2.1)	.742	1.0 (0.4-2.6)	.984
Parvalbumin	Mild	0.6 (0.4-1.0)	**.036**	0.8 (0.5-1.4)	.483
	Moderate	0.5 (0.3-0.7)	**<.001**	0.5 (0.3-0.8)	**.004**
	Severe	3.6 (2.4-5.4)	**<.001**	3.7 (2.3-6.1)	**<.001**
Sarcoplasmic calcium-binding protein	Mild	0.6 (0.2-2.2)	.479	0.9 (0.2-3.3)	.817
	Moderate	0.9 (0.4-2.4)	.908	1.0 (0.4-2.7)	.998
	Severe	4.2 (1.7-10)	**.001**	4.7 (1.7-13.3)	**.004**
Tropomyosin	Mild	0.2 (0.1-0.3)	**<.001**	0.3 (0.2-0.5)	**<.001**
	Moderate	2.9 (2.1-3.9)	**<.001**	2.0 (1.4-2.8)	**<.001**
	Severe	0.9 (0.6-1.4)	.708	1.3 (0.8-2.0)	.305
Troponin C	Mild	0.5 (0.2-1.0)	**.040**	0.6 (0.3-1.4)	.223
	Moderate	1.3 (0.8-2.2)	.260	1.3 (0.7-2.2)	.382
	Severe	0.8 (0.4-1.6)	.474	0.6 (0.2-1.3)	.186

ORs with 95% CIs are reported for both univariate and multivariable logistic regression analyses. For each severity category, a separate binary model was fitted comparing that category against all other patients. Multivariable models (OR_adj) were adjusted for age and sex. Analyses were performed on complete cases; no missing data were present. Statistically significant associations (*P* < .05) are indicated in bold. Absolute counts (n) and denominators used to compute percentages are provided in Tables I and II.

*PI*, Protease inhibitor.

After multivariable adjustment for age and sex, distinct allergen-specific patterns of association with clinical severity were observed, reflecting the underlying biological source of sensitization. Ani s 1, a parasite-derived allergen, remained independently associated with both moderate (adjusted odds ratio [OR_adj], 2.0; 95% CI, 1.3-3.3; *P* = .003) and severe reactions (OR_adj, 2.7; 95% CI, 1.5-4.8; *P* = .001), supporting the clinical relevance of *Anisakis*-related sensitization as a marker of systemic risk rather than incidental exposure.

Parvalbumin, the major fish allergen, remained independently associated with severe reactions (OR_adj, 3.7; 95% CI, 2.3-6.1; *P* < .001), reinforcing its role as a molecular indicator of clinically significant and potentially high-risk fish allergy. In contrast, tropomyosin—the principal panallergen of crustaceans and molluscs—was preferentially associated with moderate reactions (OR_adj, 2.0; 95% CI, 1.4-2.8; *P* < .001), consistent with a pattern of primary shellfish allergy or cross-reactivity across invertebrate species.

Arginine kinase, another invertebrate panallergen, was independently associated with membership in the mild category versus all other subjects (OR_adj, 5.3; 95% CI, 3.4-8.4; *P* < .001), suggesting that not all shellfish-related molecular sensitizations confer comparable clinical risk. Sarcoplasmic CBP showed the highest point estimate for severe outcomes (OR_adj, 4.7; 95% CI, 1.7-13.3; *P* = .004); however, the limited number of sensitized individuals warrants cautious interpretation of this finding. No independent associations were retained for enolase/aldolase, myosin light chain, or troponin C after multivariable adjustment.

Collectively, these data indicate that reaction severity is not uniformly distributed across patients with seafood allergy but varies according to the underlying molecular sensitization profile, with fish-associated allergens predominantly linked to more severe phenotypes and invertebrate allergens displaying a more heterogeneous severity pattern. All logistic regression models demonstrated stable convergence and adequate calibration (Hosmer–Lemeshow *P* > .05).

Sensitivity analyses excluding asymptomatic sensitized individuals yielded consistent effect estimates and did not materially alter the magnitude or direction of associations, further supporting the robustness and clinical relevance of the observed molecular–clinical relationships.

## Discussion

### Principal findings and conceptual framework

In this large multicenter cohort, multiplex molecular profiling revealed that seafood allergy is not a uniform entity but comprises discrete IgE sensitization patterns that are reproducibly associated with distinct clinical phenotypes.

Unsupervised clustering and PCA consistently identified parvalbumin, Ani s 1, tropomyosin, and arginine kinase as major contributors to molecular heterogeneity, suggesting that a single continuum of cross-reactivity may not adequately explain the observed patterns. The spatial segregation observed in multivariate analyses is consistent with distinct patterns of IgE reactivity associated with different clinical outcomes. Although mechanistic pathways were not directly interrogated, the stability of clustering across complementary unsupervised approaches and its reproducible association with clinical severity support the operational identification of IgE-defined molecular endotypes within the limits inherent to observational data.

Rather than differing only in prevalence, these molecular profiles showed divergent associations with reaction severity. Sensitization to parvalbumin and Ani s 1 was more frequently observed among subjects with severe clinical phenotypes, whereas arginine kinase sensitization was predominantly observed among subjects with mild phenotypes; tropomyosin occupied an intermediate position, being more frequently associated with moderate reactions. Importantly, these relationships were stable across complementary analytical approaches, reinforcing their statistical association with clinical severity.

Hierarchical clustering further contextualized these findings by identifying a myofibrillar-associated module comprising tropomyosin, troponin C, myosin light chain, and sarcoplasmic CBP, within which arginine kinase clustered while maintaining a distinct profile. This pattern is compatible with shared muscle-related structural characteristics combined with molecular heterogeneity.^11^, ^12^, ^13^, ^14^, ^15^ In contrast, parvalbumin clustered separately, often together with enolase and aldolase, delineating a classical fish-associated sensitization profile.^16^, ^17^, ^18^ Ani s 1 consistently segregated into an isolated cluster, consistent with a parasite-related sensitization pattern rather than cross-reactive sensitization.^19^, ^20^, ^21^

### Biological interpretation

The modular organization observed may be related to shared biochemical characteristics of the allergens and differences in exposure pathways. Parvalbumin, a heat-stable CBP abundantly expressed in fish muscle, is consistent with systemic, ingestion-mediated reactions.^17^, ^18^ Ani s 1, a parasite-derived Kunitz-type protease inhibitor, has been associated with parasite exposure, more severe systemic reactions, and occupational sensitization.^20^, ^21^, ^22^

In contrast, arginine kinase—thermolabile and known to share homology with arthropod allergens—was more frequently observed in subjects with mild clinical manifestations.^12^, ^14^, ^23^, ^24^, ^25^ Tropomyosin’s established role as a panallergen explains its broad prevalence and intermediate clinical risk, mediating cross-reactivity across crustaceans, mites, and insects without uniformly translating into severe reactions.^11^, ^25^, ^26^, ^27^, ^28^ The high point estimate observed for sarcoplasmic CBP, despite its low prevalence, underscores that rare sensitization profiles may be associated with higher point estimates for severe reactions at the population level, although wide CIs warrant cautious interpretation.^23^, ^29^

### Clinical implications

From a clinical perspective, these findings reinforce the added value of molecular allergology for risk-discriminative profiling in seafood allergy. Molecular profiles provided information beyond extract-based testing, allowing differentiation between sensitization patterns associated with markedly different clinical trajectories. Identification of parvalbumin or Ani s 1 sensitization may support a more cautious clinical assessment, whereas isolated arginine kinase sensitization may support a more conservative, individualized management strategy, pending confirmatory testing.

The limited overlap among major allergen families further suggests that indiscriminate avoidance of all seafood is often unnecessary.^4^, ^5^, ^30^, ^31^ Integrating molecular data with clinical history may contribute to more tailored dietary recommendations, in line with precision medicine approaches in allergy care^2^, ^3^, ^7^; however, such an approach should be regarded as risk-discriminative rather than diagnostic in isolation and should not replace oral food challenges when the clinical relevance of sensitization remains uncertain. The frequent observation of asymptomatic sensitization—especially to tropomyosin and arginine kinase—highlights that molecular sensitization alone does not equate to clinical risk and should be interpreted within an endotype-based framework.^32^, ^33^ This is particularly relevant in patients with overlapping sensitization patterns, in whom molecular results may indicate potential risk strata but cannot, by themselves, distinguish true clinical allergy from clinically silent IgE reactivity.

Our findings align with the broader shift toward precision allergy medicine, in which molecular diagnostics transcend extract-based categorization and support risk-informed, component-driven dietary counseling.

In patients with overlapping molecular profiles, the clinical meaning of each component may be more difficult to disentangle. Coreactivity to parvalbumin, tropomyosin, Ani s 1, or arginine kinase may reflect multiple primary sensitization events, cross-reactivity across taxonomically related or unrelated sources, or clinically irrelevant IgE binding. Therefore, these overlapping profiles should be interpreted as probabilistic risk patterns rather than as mutually exclusive diagnostic categories. In clinical practice, discordance between molecular sensitization and the reported reaction history should prompt cautious interpretation, and, when appropriate, supervised oral food challenge remains necessary to distinguish true allergy from sensitization alone.

### Strengths and limitations

Key strengths of this study include the large, prospectively recruited multicenter cohort, blinded clinical phenotyping, and the integrated use of complementary analytical approaches to interrogate molecular patterns. Several limitations should be acknowledged. First, clinical phenotypes were based on structured history rather than systematic oral food challenges, which remain the reference standard for food allergy diagnosis,^34^ particularly in patients with asymptomatic or overlapping sensitization profiles in whom history alone may not establish the clinical relevance of IgE reactivity. Although severity was classified according to validated criteria^8^ and collected prospectively, the absence of challenge-confirmed outcomes may have introduced some degree of misclassification. Therefore, the molecular profiles identified here should be viewed as hypothesis generating and clinically supportive rather than as definitive predictors of future reaction severity. Second, recruitment was conducted in tertiary referral allergy centers, potentially introducing referral bias and limiting generalizability to primary care or community populations. This setting may also have favored the inclusion of patients with more complex or polysensitized profiles, possibly leading to overrepresentation of multiple molecular reactivities. Third, geographic variability in Anisakis exposure across participating regions may have influenced parasite-related sensitization patterns, limiting extrapolation to populations with different dietary habits or parasite prevalence.^21^ Furthermore, the multiplex assay platform, although comprehensive, does not encompass all potential seafood allergens, and rare or yet uncharacterized components may not have been captured. In addition, the absence of longitudinal follow-up precludes the assessment of the temporal stability of molecular sensitization patterns and their evolution over time. Finally, the low prevalence of certain components, such as sarcoplasmic CBP, limits statistical precision and warrants cautious interpretation pending external validation. Nevertheless, reaction severity was prospectively collected using standardized criteria and analyzed independently of molecular results, mitigating the risk of differential misclassification and strengthening the internal validity of the findings. Longitudinal follow-up of this cohort, including documentation of reactions after subsequent exposures and, where ethically and clinically appropriate, supervised oral food challenges, will be required to determine whether these IgE-defined endotypes retain predictive value over time.

Taken together, these limitations do not undermine the internal consistency of the clustering architecture observed, although external validation in longitudinal and challenge-confirmed cohorts remains necessary before these molecular patterns can be broadly implemented as tools for individualized prediction or dietary decision-making.

### Conclusion

Seafood allergy is organized into distinct molecular endotypes defined by IgE sensitization to parvalbumin, Ani s 1, tropomyosin, and arginine kinase, each associated with a characteristic clinical risk profile.

Endotype-based interpretation of molecular sensitization extends beyond extract-level diagnostics and supports clinically meaningful risk-discriminative profiling and personalized dietary counseling.

## Disclosure statement

Supported by the “Progetto Ricerca Corrente” of the Italian Ministry of Health, Rome, Italy.

Disclosure of potential conflict of interest: E. Scala has received consultant arrangements and participated in speakers’ bureaus for Dasit and Thermo Fisher Scientific. The rest of the authors declare that they have no relevant conflicts of interest.
